# Total spine magnetic resonance imaging for detection of multifocal infection in pyogenic spondylodiscitis: a retrospective observational study

**DOI:** 10.1186/s12891-020-03928-5

**Published:** 2021-01-14

**Authors:** Jeanette Henkelmann, Timm Denecke, Philipp Pieroh, Stephanie Einhorn, Nicolas H. von der Hoeh, Christoph-Eckhard Heyde, Anna Voelker

**Affiliations:** 1 Department for Diagnostic and Interventional Radiology, University Hospital Leipzig, Liebigstraße 20, 04103 Leipzig, Germany; 2 Department for Orthopedics, Trauma and Plastic Surgery, University Hospital Leipzig, Liebigstraße 20, 04103 Leipzig, Germany

**Keywords:** Pyogenic spondylodiscitis, Multifocal infection, Spine, Infection, Magnetic resonance imaging

## Abstract

**Background:**

Due to the unspecific symptoms of spondylodiscitis (SpD), an early radiological examination is necessary. However, controversially discussed is the need for magnetic resonance imaging of the entire spine to exclude multisegmental infections and to determine the required surgical interventions. The aims of this study were to assess the incidence of multilevel non-contiguous pyogenic SpD and compare comorbidities, pain symptoms, and subsequent surgical strategies between unifocal (uSpD) and multifocal (mSpD) SpD.

**Methods:**

We retrospectively evaluated the data of patients with confirmed, surgically treated, pyogenic SpD who had received a total spine MRI in a single spine center between 2016 and 2018. MRI findings were classified according to Pola-classification and demographics, duration of clinical symptoms (pain and neurology) and Charlson Comorbidity-Index (CCI) results were compared between uSpD und mSpD groups. Surgical therapy was evaluated in patients with mSpD.

**Results:**

uSpD was detected by MRI in 69 of 79 patients (87%). Of these, mSpD was detected in 10 patients (13%) with 21 infected segments (cervical and/ or thoracic and/ or lumbar region). Age and CCI were similar between uSpD and mSpD and 24 of all SpD regions were clinically unapparent. All patients with uSpD were treated operatively. In seven patients with mSpD, all infected levels of the spine were treated surgically in a one-stage procedure; one patient had a two-stage procedure and one patient had surgery at the lumbar spine, and an additional infected segment of the upper thoracic spine was treated conservatively. One patient died before a planned two-stage procedure was performed.

**Conclusions:**

Due to mSpD being found in approximately 13% of SpD cases, and considering the risk of overlooking an mSpD case, MRI imaging of the total spine is recommended. The detection of multiple infection levels can have an impact on the therapeutic strategy chosen.

## Background

The incidence of pyogenic spondylodiscitis (SpD) (0.2–2.4 cases per 100,000 people per year) has risen, probably due to increases in life expectancy and associated comorbidities [1]. Pyogenic SpD occurs predominantly in the lumbar spine (58%), followed by the thoracic spine (30%) and cervical spine (11%) [2]. The non-specific clinical appearance impairs the diagnosis, especially in patients with pre-existing degenerative spinal disorders [3]. Magnetic resonance imaging (MRI) is the gold standard for pyogenic SpD diagnostic imaging due to its high specificity (96%) and sensitivity (92%). Typical pyogenic SpD associated MRI findings include, hyperintense intervertebral disc and/or adjacent vertebra bodies in fat suppressed T2 -weighted (w)/ short tau inversion recovery (STIR) sequence and hypointense intervertebral disc and/or bodies in T1-weigted-sequence [4]. Decreased disc height, end plate erosions or destructions, signs of paraspinal and epidural inflammation and epidural or psoas abscesses can also be detected [5]. The Pola classification considers these MRI morphological aspects of SpD as well as a possible neurological deficit and is thus a possible grading system for the severity of SpD [6]. In the case of tuberculous SpD, the entire spinal column should be imaged during diagnosis since additional silent foci are often present [7]. In contrast, literature regarding the widening of MRI imaging, from local to total spine imaging, in cases of pyogenic SpD is controversial. Although in a few references it is discussed that in up to 30% of cases with pyogenic SpD multiple foci were observed [5, 8, 9], data are inconsistent as to whether the affected levels are contiguous or non-contiguous or if they occur in different regions of the spine. One study found that 13% of spinal infections in patients with pyogen and specific (e.g. tuberculosis) SpD were non-contiguous [10]. Although these data highlight the opportunity to miss a non-contiguous SpD, recommendations for which cases an MRI of the entire spine should be performed are missing. The primary aim of the study was to detect the frequency of unifocal (uSpD) and multifocal (mSpD) SpD in surgically treated SpD patients who had all an entire MRI of the spine. Secondarily, we compared patients with unifocal (uSpD) and multifocal (mSpD) SpD, regarding comorbidities, clinical symptoms, neurology to reveal risk factors underlining the need for an entire spine MRI to exclude non-contiguous SpD.

## Methods

### Patient population

The study was approved by the local ethics committee (427/18-ek) and was performed in accordance with the Declaration of Helsinki. In this single spine center study, we retrospectively evaluated data from patients between 2016 and 2018 with a surgically treated pyogenic SpD and a previous MRI of the entire spine. According to the in-hospital standard operating procedure for SpD all patients receive an MRI of the entire spine except contraindications are present. Inclusion and exclusion criteria are summarized in Table 1.

**Table 1:** Inclusion and exclusion criteria

292 Patients with pyogenic spondylodiscitis were treated operatively from 2016 to 2018	
Case Selection	
Inclusion:	Exclusion:
• First detection of pyogenic Spondylodiscitis with surgical treatment	• only region specific (lumbar, thoracic, cervical) MRI
• Age ≥ 18	• Contraindications for MRI
• MRI of the total spine	• Medical history of a previous Spondylodiscitis
	• Previous Spine surgery
	• Pregnancy
**79 patients with pyogenic spondylodiscitis and MRI of the total Spine**	

The diagnosis of SpD was confirmed by surgical or computer tomography-guided biopsy (microbiological and/or histological proven) and by an assessment of the clinical course combined with MRI findings.

The decision to administer surgical therapy was made depending on perioperative risk, extension of osseous destruction of the involved segment, segmental stability, degree of surrounding tissue affected, and/or the presence of an epidural abscess.

Surgical treatment could include intervertebral disc debridement, pedicle screw fixation, decompression, abscess evacuation, psoas drainage or interbody fusion. In our clinic, the dorsal surgical procedure was primarily performed in cases with lumbar or thoracic SpD, with stabilization and debridement of the disc space and an additional intersomatic fusion, using the transforaminal lumbar interbody fusion (TLIF) -technique. For larger defects in the ventral column, an addition ventral stabilization with cage or bone grafts was practiced, as described in von der Hoeh et al. [11]. In cases of SpD of the cervical spine, surgery was done as anterior cervical decompression and fusion (ACDF) as well as an additional plating. ACDF combined with additional plating shows a better fusion rate then ACDF alone in cases of cervical SpD [12]. A similar therapeutic procedure was performed for the mSpD group.

Additionally, all patients were treated with an antibiotic for 2-6 weeks post-surgery.

### Total spine magnetic resonance imaging

Total spine MRI examination was performed before surgery over one to two sessions; a two stage MRI was frequently required when the patient was assigned from an outside hospital. MRI scans were performed over two sessions for 17% of patients. The MRI was then reviewed in detail for the presence of multilevel SpD by a board-certified radiologist with 6 years’ experience specializing in musculoskeletal radiology. This review was done using the software Syngo (Siemens medical solutions, Germany). MRI data were analyzed and classified according to the Pola classification criteria solely to analyze the severity of SpD and not as decision criterion for surgery. Pola classifies SpD into three main groups: A - cases without neurological deficit or biomechanical instability; B – cases with significant bone destruction or biomechanical instability without neurological deficit; C – all cases with neurological deficit or the presence of epidural abscess.

All MRI scans were performed using a 1.5 Tesla MRI (Aera, Siemens, Erlangen, Germany) or 3.0 Tesla MRI (Siemens Trio, Siemens or Philips Ingenia, Best, The Netherlands), with a dedicated spine coil, using a sagittal inversion recovery sequence (short tau inversion recovery (STIR) resp. turbo inversion recovery magnitude (TIRM)). Then, all affected spinal segments with a focus suspected of infection, which was detected by STIR/TIRM-signalhyperintensity, the protocol was completed with the subsequent sequences: sagittal and axial and T2w turbo spin-echo sequence, sagittal T1w turbo spin-echo sequence pre- and post-contrast Gadolinium and axial T1w fat-saturated post-contrast. All affected SpD segments were completely imaged on MRI with contrast enhanced protocol to determine the exact extent in the affected segment.

### Analyzed parameters

The primary outcome of the study was detecting the presence or absence of non-contiguous mSpD.

The secondary outcomes were the investigation and analysis of epidemiological factors and comorbidities in patients with uSpD and mSpD as well as the analysis of the affected region and levels.

Demographics, length of hospital stay, readmission rate, mortality and comorbidity severity were assessed using the Charlson Comorbidity Index (CCI). Neurological deficit severity was graded according to the American Spinal Injury Association (ASIA) scale.

The duration of clinical symptoms (pain and neurological deficit) before hospital admission and the surgical strategies used, depending on the spine segment involvement (uSpD vs. mSpD) were also analyzed. Pain history was analyzed using the statement “painful yes/no?” according to segment localization (cervical, thoracic, lumbar) of SpD in MRI. Microbiology samples (e.g. blood cultures, intraoperative samples) were also compared.

### Statistical analysis

Graphs and analyses were generated using Graph Pad Prism software 7 (GraphPad software, La Jolla, USA). Data are presented as mean ± standard deviation. The age distribution was analyzed using the Shapiro-Wilk-test, yielding a non-Gaussian distribution. Thus, data were analyzed using the Mann-Whitney test. Differences in gender were analyzed with the Fisher exact test and the CCI was compared with the Kolmogorov-Smirnov test. The level of significance was defined as *p* < 0.05.

## Results

Sixty-nine patients suffered from uSpD and ten from mSpD. Descriptive data are shown in Table 2. There was no significant difference in age or CCI between the groups except regarding to the subgroup COPD shown in Table 3.

**Table 2:** Descriptive Data of all investigated Patients with Spondylodiscitis (SpD)

	Unifocal SpD	Multifocal SpD	*p* -value
No. of patients	69	10	
Gender (ratio male:female)	46:23	7:3	> 0.99
Age (years, mean ± standard deviation)	70.28 ± 11.93	71.39 ± 11.62	0.81
Charslon Comorbidity-Index	4.61 ± 2.35	4.5 ± 1.72	0.98
infected region (amount [n])	cervical: 7	cervical: 6	
	thoracic: 14	thoracic: 7	
	lumbar: 48	lumbar: 8	
Pain at the infected regions (yes:no)	57: 12	9: 12

**Table 3:** Subgroup analysis of Charlson Comorbidity Index

Subgroups CCI	Unifocal	Multifocal	*p*-Value
	*Y/N*	*Y/N*	
COPD	8/61	4/6	p = 0.0401*
Nothing	18/51	0/10	*p* = 0.1056
Myocard Infarction	8/61	3/7	*p* = 0.1397
Peripheral Vascular Disease	10/59	0/10	*p* = 0.3455
Diabetes without complications	17/52	4/6	*p* = 0.4430
Solid Tumor without metastases	5/64	1/9	*p* = 0.5687
Moderate/severe Kidney Failure	13/56	1/9	*p* = 0.6812
Cerebrovascular Disease	3/66	0/10	*p* > 0.9999
Connective Tissue Disease	0/69	0/10	*p* > 0.9999
Peptic Ulcer Disease	0/69	0/10	*p* > 0.9999
Mild Liver Disease	1/68	0/10	*p* > 0.9999
Dementia	1/68	0/10	*p* > 0.9999
Diabetes with organ damage	8/61	1/9	*p* > 0.9999
Hemiplegia	0/69	0/10	*p* > 0.9999
Leukemia	0/69	0/10	*p* > 0.9999
Lymphoma	1/68	0/10	*p* > 0.9999
Moderate/severe Liver Disease	4/65	0/10	*p* > 0.9999
Solid Tumor with metastases	2/67	0/10	*p* > 0.9999
AIDS	0/69	0/10	*p* > 0.9999
Chronic Heart Failure	19/50	2/8	*p* > 0.9999

In patients with uSpD, the lumbar spine was most frequently affected (69.5%), followed by the thoracic spine (20.3%) and cervical spine (10.2%).

In the mSpD cases a total of 21 spinal segments were infected; no spinal region showed a dominant frequency over the others (lumbar: 8, thoracic: 7, cervical spine: 6). However, four infection patterns in mSpD were detected: cervical + thoracic (*n* = 2), cervical + lumbar (*n* = 3), lumbar + thoracic (*n* = 4) and cervical + thoracic + lumbar (*n* = 1).

**Fig. 1:**
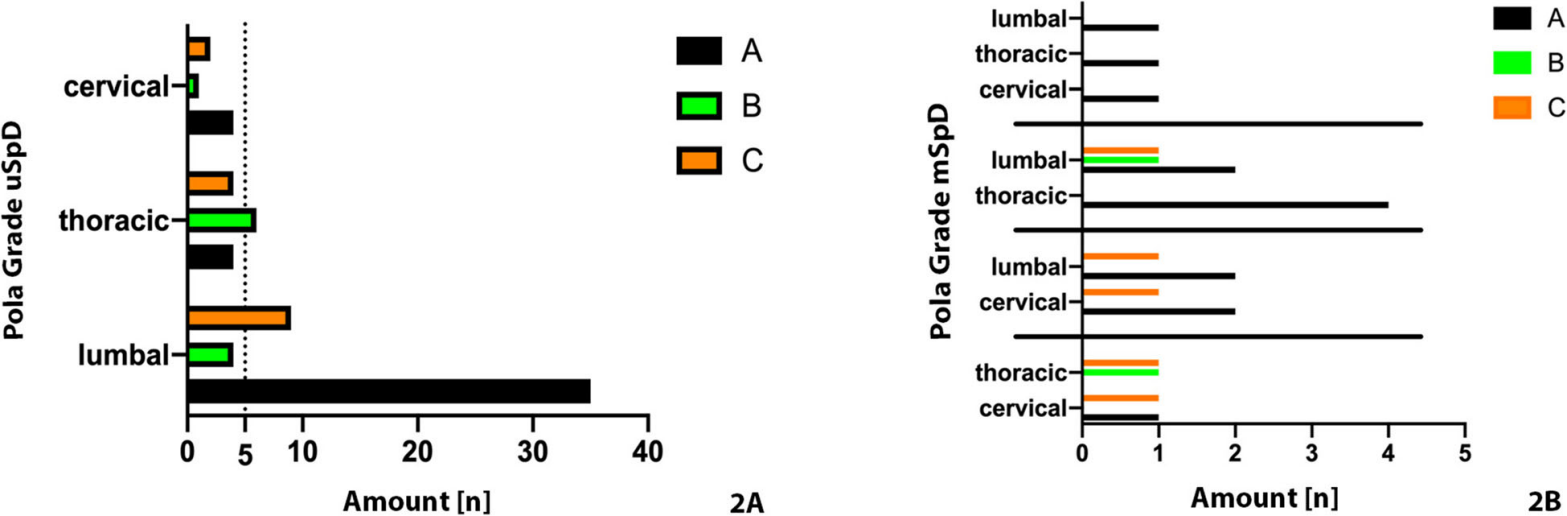
Pola classification for the affected SpD segments regarding to the involved region in unifocal (**a**) and multifocal (**b**) spondylodiscitis

Pola classifications in the unifocal group were distributed as follows: Type A, *n* = 43; type B, *n* = 11 and type C, *n* = 15. The specific Pola-Classification distribution is shown in Fig. 1a. The Pola classification distributions were similar for mSpD cases: Type A (*n* = 13) then Type B (*n* = 2) and Type C (*n* = 5) (Fig. 1b). Thus, no difference in severity according to the Pola classification between uSpD and mSpD were detected.

Most patients had symptoms (pain, neurological deficit, septic shock or organ failure) onset 1 week (*n* = 31 uSpD, *n* = 4 mSpD) or 1 to 2 months (*n* = 34 uSpD, *n* = 5 mSpD) before admission. Long-term symptoms (3 to 6 months) were reported for four patients with uSpD and for one patient with mSpD and one Patient with uSpD had a history of symptoms longer than 6 months.

In 24 of 90 affected SpD segments, no pain symptoms were reported (Table 2). Patients with lumbar uSpD (ratio pain/ no pain lumbar: *n* = 44:4) were more likely to experience pain in the specific area than patients with lumbar mSpD (*n* = 4:4). Patients with thoracic and cervical SpD had less pain in the infected area than lumbar SpD, regardless of group (ratio pain/ no pain thoracic: *n* = 9:5 uSpD, *n* = 2:5 mSpD; cervical: *n* = 4:3 uSpD, *n* = 3:3 mSpD).

Six patients with uSpD (each *n* = 1 ASIA A and D, each *n* = 2 ASIA B and C) had neurological deficits; no neurological deficits were reported in the mSpD group.

Microbiological samples from blood culture or intraoperative samples were positive in 60.9% of uSpD patients and in 60% of patients with mSpD.

The following pathogens in uSpD were found in a decreasing frequency: *Staphylococcus aureus* (*n* = 18, 42.9%), *Escherichia coli* (*n* = 8, 19.1%), *Staphylococcus epidermidis* and *Staphylococcus agalactica* (each *n* = 2, 4.8%). In patients with mSpD *S. aureus* (*n* = 5, 83.3%) and *E. coli* (n = 1, 16,7%) were detected.

Multiple pathogens were detected in three cases in the uSpD group (*n* = 1 *S. aureus* + *E. coli*, *n* = 1 *S. aureus* + *Pseudomonas aeruginosa*, *n* = 1 *Streptococcus anginosus* + *Streptococcus sanguinis*.

All patients (100%) suffering from uSpD underwent surgery and eight of the 10 patients (80%) with mSpD had surgical treatment of all infected areas of the spine. Of the mSpD patients, we performed a one-stage procedure on all infected spinal regions for seven patients (70%). However, one patient died after lumbar spinal surgery and so the planned second surgery on the cervical and thoracic spinal regions could not be performed. One patient had a two-stage procedure. One other patient underwent surgery on the lumbar spine while the infected areas at the upper thoracic spine without an epidural abscess were treated conservatively.

**Fig. 2:**
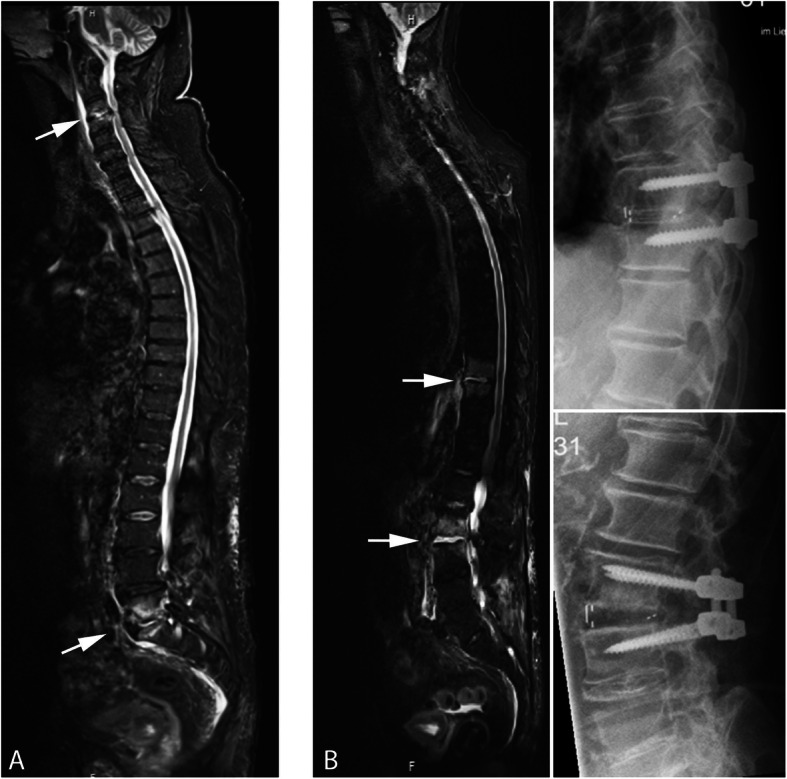
MRI with Short Tau Inversion Recovery (STIR) sequence of the total spine (**a**) in a 75–80-years old patient with mSpD in the segment C3/4 and L5/S1. Arrows show the typical hyperintensity signal alterations in the disc and adjacent endplates with a slightly prevertebral fluid collection (asterisk). **b**: MRI of an 80–85-years old patient with mSpD in the segment Th9/10 and L2/3 and postoperative X-ray after posterior short segment stabilization and intersomatic fusion with cage in TLIF-technique in both levels

Figure 2 shows two examples of mSpD with total spine MRI and one postoperative follow-up X-ray.

The mean duration of hospital stay was 29 days ±14 days for uSpD patients and 45 days ±27 days for mSpD patients. The differences in duration was not statistically significant. The uSpD readmission rate was 11.6% and there were no readmissions from the mSpD group.

The mortality rate during hospital stay was 11.6% (*n* = 8) in patients with uSpD and 10% (*n* = 1) in patients with mSpD.

## Discussion

In the present study the frequency of mSpD was 12.7% and there were no differences in the clinical presentation, symptoms, morbidities to patients suffering from uSpD. However, the absence of pain in the affected region especially in the thoracic and cervical region highlight the need for an entire spine MRI to exclude non-contiguous SpD. Currently, for pyogenic SpD there is no clear recommendation for whole spine MRI to exclude non-contiguous infected segments. MRI is a non-invasive, non-ionising imaging technique.

It is well known that patients with tuberculous SpD can have non-contiguous infected segments of the spine. We also know that cervical and thoracic involvement is more frequent in tuberculous SpD then in patients with unspecific SpD [13]. For this reason, it is undisputed that in patients with tuberculous SpD, the entire spinal column should be imaged by MRI, even if the patients have no pain at other levels of the spine [14]. In a study by Abbara et al. [14], they could not detect mSpD in patients with pyogenic SpD and so did not recommend total spine MRI in patients with unspecific SpD. In response to this study, Siam et al. discussed the study population of Abbara, and pointed out that, the study size of 19 cases of unspecific SpD is too small and noted that it was not reported whether the patients had had a total spine MRI. As a result, Siam et al. recommend a whole spine MRI be performed for all patients with SpD [7].

Two studies have evaluated the percentage of multiple non-contiguous infected segments as 6.8% [15] and 13% [10] in patients with pyogenic SpD. Our data showed that 12.7% of pyogenic SpD cases were mSpD. Taken together, these high numbers of mSpD in all three studies support the conclusion that an MRI of the entire spine must be strongly recommended.

Similar to previous studies our patient cohort was geriatric (mean age uSpD = 70 years, mSpD = 71 years) and men were more frequently affected [16] but there was no evidence for a higher frequency of mSpD due a higher age.

Although it is well known that SpD is more common in patients with secondary diseases such as diabetes and renal insufficiency [17], it is unclear whether mSpD is associated with the severity of comorbidities. In the present study, we measured the severity of comorbidities via the CCI.

The CCI of patients with uSpD and mSpD were similar. In this light, it seems rational to examine all SpD patients for mSpD, as there does not appear to be an easy metric for distinguishing between the two groups. Although generally the number and severity of comorbidities are linked to poorer patient outcomes, our data contradict the hypothesis that there is a higher risk of mSpD associated with these factors [18, 19].

Back pain is the cardinal symptom of SpD [20]. Shiban et al. [21] reported back pain in 100% of SpD patients and Valencius [22] in 72% of SpD patients. In our cohort 85% (*n* = 57) of patients with uSpD reported pain. However, in the mSpD group only 20% patients indicated pain at all infected spinal regions.

**Fig. 3:**
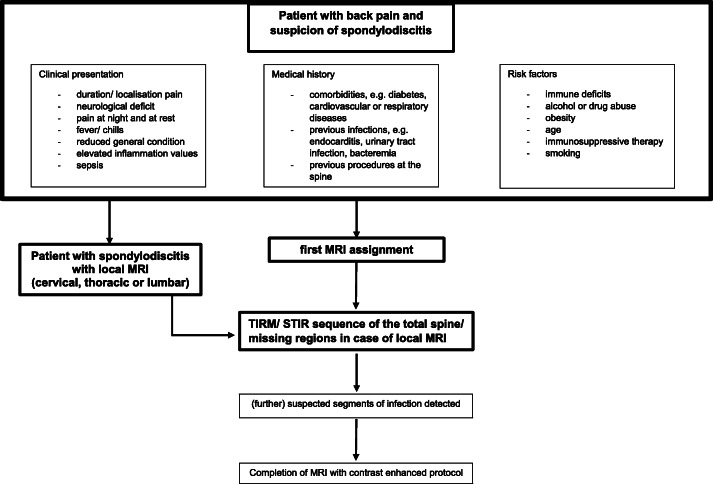
It is shown an algorithm that provides anamnestic indicators of possible spondylodiscitis in patients with back pain. In addition, it shows a possible diagnostic radiological pathway in case of suspected spondylodiscitis

Therefore, we recommend that local MRI to detect SpD should be extended to the entire spine. However, it must be taken into account that other non-infectious pathologies may be detected as well. Radiologists and referring clinicians need to be aware of potential SpD mimics including Modic Type I degenerative changes and other pathologies such as trauma or metastatic disease [23–25]. For correct diagnosis it is necessary to know the specific patterns of each of these diseases and be able to distinguish them from infectious SpD. Not least, it also remains the task of radiology to evaluate image acquisition during the scan and to adapt the sequence protocol, including the recommendation to image the total spine when a focus of infection is detected (Fig. 3).

An additional cost factor results merely from the increased measurement time, usually not from patient repositioning or additive contrast agent. The extended MRI time is a critical point that must be taken into account when focusing on limited MRI resources. On the other hand, the relevant gain in information must be considered, especially in light of the fact that an early detection and a possible early therapy start is crucial for the outcome in case of an SpD infection.

Only patients in the uSpD group (8.7%, ASIA A-D) presented with a neurological deficit at admission. The literature gives contradictory information in this regard. In some studies, up to 54% of cases with SpD have been associated with functional neurological deficits [21, 26]. However, there are also studies with a smaller number of cases that associate SpD with extremity weakness (22%) which is more consistent with our results in this study [27]. This could be explained by the fact that SpD was detected in our patients at an earlier stage than in previous reports. Since none of the patients in the mSpD group had a neurological deficit, this symptom does not appear to be indicative of mSpD. Thus, neurological deficits do not appear to indicate the involvement of multiple spinal regions in SpD.

The short time from symptom onset to clinical admission is a likely reason for the low incidence of neurological deficits in our cohort.

There is no common classification to categorize the image changes in SpD. However, Pola et al. published a simple classification based on MRI images of SpD in 2017 [6]. We used the Pola-Classification to classify the patients in our study as all study participants had an MRI. In our study we found more cases with Pola-A (62%) and less cases with Pola-C (22%) compared to the published study group from Pola et al. (Pola-A 33,6%, Pola-B 18,4%, Pola C-48%) [6]. We believe that this is because our patients were likely diagnosed with SpD at an earlier stage than in the cited publication.

In Patients with mSpD we found the following Pola Classification distribution pattern: *n* = 21 (all affected segments); Pola-A *n* = 14; Pola-B n = 2, Pola-C *n* = 5. This shows that a multifocal presentation of SpD does not seem to be associated with a higher degree of severity with regards to the Pola-Classification and is not associated with more epidural abscesses. Another reason for our results could be that we only included patients with primary SpD without previous surgery on the spine. This could explain why there are fewer epidural abscesses in our data compared to Pola et al. [28].

In the 2018 published study, 23% of patients included had post-surgery SpD. Furthermore, the missing neurological deficits and bony destruction seems to be responsible for a large part of the missing classification upgrade.

We performed surgery in eight cases (80%) with mSpD, on all involved spinal regions, and in seven of eight cases as a one-stage procedure. One patient with involvement of all three areas of the spine died after surgery of the lumbar spine, preventing completion of the two-session procedure that had been planned. One patient had a two-session procedure. One other patient underwent surgery only on the lumbar spine while the infected areas at thoracic spine were treated conservatively. All patients were treated with an antibiotic post-surgery. This therapeutic strategy of early surgical therapy and antibiotics has also been supported by Tsai et al. [29]. This study reported better outcomes for patients with SpD who were treated with early surgery together with antibiotics compared to antibiotics alone including shorter hospital stays, improvement in kyphotic deformities and increased quality of life. For findings requiring a conservative approach to treatment, we recommend a follow-up MRI for an evaluation of the therapy response and, if necessary, a change of therapy.

Nevertheless, the current study has some limitations. First, it is a retrospective study. As such, the decision on which treatment to pursue was not random.

A follow-up to evaluate the outcome was not the focus of this study. It was concerned with the decision of the therapeutic approach at the time of imaging.

There are also many confounding factors, such as the degree of infection, the duration of treatment and the surgical procedure, which can influence the outcome results. This work focused on the practical approach to evaluating the final diagnosis.

## Conclusions

In our patient cohort, 13% were diagnosed with mSpD. Pain history did not appear to be indicative of SpD level. Furthermore, no difference in age and CCI could be found in between the uSpD and mSpD groups. MRI images classified by Pola-classification showed that both groups were predominantly classified as Pola-A.

This study focuses on the necessity to capture all potential foci. The risk of missing an additional infectious spinal focus may worsen patient outcomes and might require multi-operative management. Due to the missing of a higher index for comorbidities for mSpD, there is currently no obvious predictor for mSpD, MRI of the entire spinal column should be performed for each patient with suspicious SpD.

## Data Availability

The datasets used during the current study are available from the corresponding author on reasonable request.

## Abbreviations

ACDF: anterior cervical decompression and fusion
CCI: Charlson Comorbidity-Index
m: Multifocal
MRI: Magnetic Resonance Imaging
SpD: Spondylidiscitis
STIR: Short Tau Inversion Recovery
TIRM: Turbo Inversion Recovery Magnitude
TLIF: Transforaminal lumbar interbody fusion
u: Unifocal
